# Collateral Impact of COVID-19 Prevention Measures on Re-Emergence of Scarlet Fever and Pertussis in Mainland China and Hong Kong China

**DOI:** 10.3390/ijerph19169909

**Published:** 2022-08-11

**Authors:** Yiran He, Chenjin Ma, Xiangyu Guo, Jinren Pan, Wangli Xu, Shelan Liu

**Affiliations:** 1 Center for Applied Statistics, School of Statistics, Renmin University of China, Beijing 100872, China; 2 College of Statistics and Data Science, Faculty of Science, Beijing University of Technology, Beijing 100124, China; 3 Department of Infectious Diseases, Zhejiang Provincial Center for Disease Control and Prevention, Hangzhou 310051, China

**Keywords:** scarlet fever, pertussis, COVID-19, Mainland China, Hong Kong

## Abstract

The incidence of scarlet fever and pertussis has increased significantly in China in recent years. During the COVID-19 pandemic, stringent non-pharmaceutical intervention measures were widely adopted to contain the spread of the virus, which may also have essential collateral impacts on other infectious diseases, such as scarlet fever and pertussis. We compared the incidence data of scarlet fever and pertussis in Mainland China and Hong Kong from 2004 to 2021 before and after the COVID-19 pandemic. The results show that the incidence of both diseases decreased significantly in 2020–2021 compared to the after-re-emergence stage in these two locations. Specifically, in 2020, scarlet fever decreased by 73.13% and pertussis by 76.63% in Mainland China, and 83.70% and 76.10%, respectively, in Hong Kong. In the absence of COVID-19, the predicted incidence of both diseases was much higher than the actual incidence in Mainland China and Hong Kong in 2020–2021. This study demonstrates that non-pharmaceutical measures implemented during the COVID-19 pandemic can partially reduce scarlet fever and pertussis re-emergence in Mainland China and Hong Kong.

## 1. Introduction

Scarlet fever and pertussis are acute respiratory infectious diseases that are highly contagious and spread mainly through air and droplets. In general, people are vulnerable to infection, but children are more susceptible. After decades of a consistently low incidence of scarlet fever and pertussis, a dramatic increase has been seen in many countries in recent years, such as the United Kingdom [1,2], China [3,4], and Korea [5,6]. Scarlet fever is an acute respiratory infection that develops in some people who have strep throat. In recent years, due to a lack of vaccines, the widespread use of antibiotics, drug resistance, and pathogen diversity, the re-emergence of this disease has become a great threat to global health, as has been seen in Korea and Hong Kong [6,7]. Increased cases of scarlet fever have also been reported in Mainland China [8]. Pertussis, another bacterial illness caused by *Bordetella pertussis*, was among the leading causes of infant mortality worldwide and remains a public health problem today [9]. Since the 1990s, concentrated outbreaks of pertussis have been reported in developed countries with high vaccine coverage, such as the United States, the United Kingdom, and Australia, with a significant increase in cases among adolescents and adults. This phenomenon is called pertussis re-emergence [10]. In Mainland China, the incidence of pertussis has increased significantly in some cities, such as Tianjin, which shows the characteristics of pertussis re-emergence [11]. The Shandong and Chongqing Provinces have also experienced local outbreaks or epidemics of pertussis in recent years [12,13]. In Hong Kong, the number of reported cases of pertussis has surged since 2017, with 110 cases reported in 2018, reaching the highest number in recent decades [14]. Stopping the spread of scarlet fever and pertussis remains a public health priority.

Coronavirus disease 2019 (COVID-19) is caused by severe acute respiratory syndrome coronavirus 2 (SARS-CoV-2). The first case in Mainland China was identified in Wuhan in December 2019 [15], and the number of infected has skyrocketed since then. In Hong Kong, the first case of COVID-19 was announced on 23 January 2020 [16]. As of 15 March 2022, a total of 458,651,513 confirmed cases of COVID-19 have been reported to the World Health Organization globally, including 6,048,995 deaths [17]. To control the large-scale outbreak and fast spread of COVID-19, China applied strict containment and suppression strategies in the early stage of the outbreak, which resulted in suspending businesses, schools, and transportation, strengthening entry and exit control, canceling large-scale events, avoiding personal exchanges and contact, and isolating and treating patients and suspected patients in situ. On 23 January 2020, Wuhan, Hubei Province in Mainland China, implemented lockdown measures. On 29 January, 31 provinces launched a level-I public health emergency response in Mainland China. Under strict containment and suppression preventive measures, the number of confirmed and suspected cases of COVID-19 in China decreased drastically. As reported, China brought the epidemic under control for the first time at the end of March [18], and most Chinese provinces had adjusted their public health emergency response from level I/II to level III/IV by mid-March 2020, when people were allowed to return to work and gradually resumed their daily activities. As of 29 April 2020, epidemic prevention and control had dropped to the level of routine strategies, such as wearing face masks and using health codes [18]. In response to the COVID-19 outbreak, Hong Kong raised its response level to the highest level of emergency on 25 January 2020 and adopted appropriate prevention and control measures to contain the development of the epidemic [19]. Public services were gradually restored in Hong Kong on 4 May, as the situation gradually improved [20]. According to the Chinese government’s categorization of COVID-19 stages, the period January–April 2020 was identified as the emergency stage of COVID-19 in China, and the period May–December 2020 was identified as its routine stage. 

Non-pharmacological intervention measures, such as social distancing control, personal movement restriction, and strengthening of personal protective measures, have effectively reduced the spread of COVID-19. These measures have played an important role not only in the prevention and control of COVID-19 but also in the impact on the incidence of other infectious diseases. For example, in Jiangsu Province, China, tuberculosis notifications dropped by 52% in 2020 compared to 2015–2019 [21]. Hu et al. reported that seasonal peaks for six respiratory infectious diseases disappeared in 2020–2021 due to public health measures in China [22]. A total of 12 notifiable communicable diseases and five non-communicable respiratory diseases decreased by 52.3% in Pakistan in 2020 compared to 2019 [23]. The incidence of influenza has declined worldwide, as seen in China, the United States [24], New Zealand [25], Japan [26], and Korea [27].

Scarlet fever and pertussis have similar modes of transmission as COVID-19 (i.e., through respiratory or contact routes). To control and prevent the COVID-19 epidemic, the Chinese government has implemented strict non-pharmacological intervention measures, providing us with research on the impact of non-pharmacological intervention measures on the incidence of scarlet fever and pertussis at different epidemic stages. However, according to our literature review, the effects of different phases of non-drug intervention policies on scarlet fever and pertussis, particularly in China, have not been well studied.

In this study, we aim to investigate and quantify the impact of a series of non-pharmacological COVID-19-related interventions on the incidence of scarlet fever and pertussis in Mainland China and Hong Kong.

## 2. Materials and Methods

### 2.1. Data Source and Collection

Monthly data on cases of scarlet fever and pertussis from 2004 to 2021 were collected from the following sources: (1) the case data in Mainland China were from the official website of the National Health Commission of the People’s Republic of China (http://www.nhc.gov.cn/jkj/s2907/new_list.shtml (accessed on 1 March 2022)); (2) the case data in Hong Kong were from the official website of the Centre for Health Protection, the Government of Hong Kong Special Administrative Region (https://www.chp.gov.hk/sc/static/24012.html (accessed on 1 March 2022)); (3) the population data of Mainland China were obtained from the China statistical yearbook published annually by the National Bureau of Statistics of the People’s Republic of China (http://www.stats.gov.cn/tjsj/ndsj/ (accessed on 1 March 2022)); (4) the population data of Hong Kong were obtained from the Hong Kong annual digest of statistics published annually by the Census and Statistics Department, the Government of Hong Kong Special Administrative Region (https://www.censtatd.gov.hk/sc/EIndexbySubject.html?pcode=B1010003&scode=460 (accessed on 1 March 2022)).

In general, the data in this study were from open websites and official statistics released by the Chinese government. It should be noted that deaths from scarlet fever and pertussis were basically zero, so we excluded the number of fatalities from this study.

### 2.2. Statistical Analysis

The monthly and yearly incidences of scarlet fever and pertussis (per 100,000 people) were defined by dividing the number of monthly and yearly cases by the population size. To visually demonstrate the impact of COVID-19 on the incidence of scarlet fever and pertussis, we classified the data into different stages: 2004–2019 (before-COVID-19) and 2020–2021 (after-COVID-19). In the before-COVID-19 stage, according to the time of re-emergence of scarlet fever and pertussis, we considered the before-re-emergence stage (scarlet fever, 2004–2010; pertussis, 2004–2016) and the after-re-emergence stage (scarlet fever, 2011–2019; pertussis, 2017–2019). The two-proportion Z-test was used to compare the changes in the incidence of scarlet fever and pertussis in the before- and after-COVID-19 stages. The Pearson correlation coefficient was employed to calculate the correlation between scarlet fever, pertussis, and COVID-19. Differences were considered statistically significant at *p* < 0.05, and all tests were two-tailed. Based on 2004–2019 morbidity data, we used the autoregressive integrated moving average model (ARIMA model) to predict the incidence of scarlet fever and pertussis in 2020–2021 [28], where COVID-19 was assumed to be absent. The ARIMA model can be expressed as ARIMA $\left(p,d,q\right)\times {\left(P,D,Q\right)}_{S}$. The parameters *p*, *d*, and *q* represent the order of non-seasonal autoregressive (AR), difference, and moving average (MA), respectively; *P*, *D*, and *Q* are corresponding seasonal orders; and *S* is the seasonal period of the data (12 months in this study). The quality of the ARIMA model is high when treating trends, seasonality, error structure, and the influence of past outbreaks. Since there were too many zeros in the number of pertussis cases in Hong Kong, first-order smoothing was performed on the incidence of pertussis in Hong Kong before predictive analysis from the ARIMA model.

We used Excel 2019, GraphPad Prism 9 (San Diego, CA, USA), and R statistical software (Vienna, Austria) for the data analysis. The ARIMA model was constructed using the function “arima” in the stats package in R statistical software.

## 3. Results

### 3.1. Description of the Incidence of Scarlet Fever and Pertussis in Mainland China

**Figure 1:**
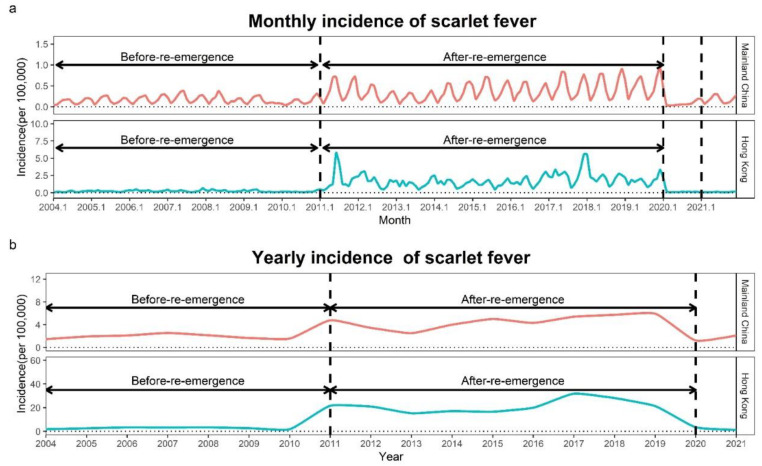
Yearly and monthly incidence rates (1/100,000) of scarlet fever in the period 2004–2021. (**a**) Monthly incidence of scarlet fever; (**b**) yearly incidence of scarlet fever. Before-re-emergence: 2004–2010; after-re-emergence: 2011–2019.

**Figure 2:**
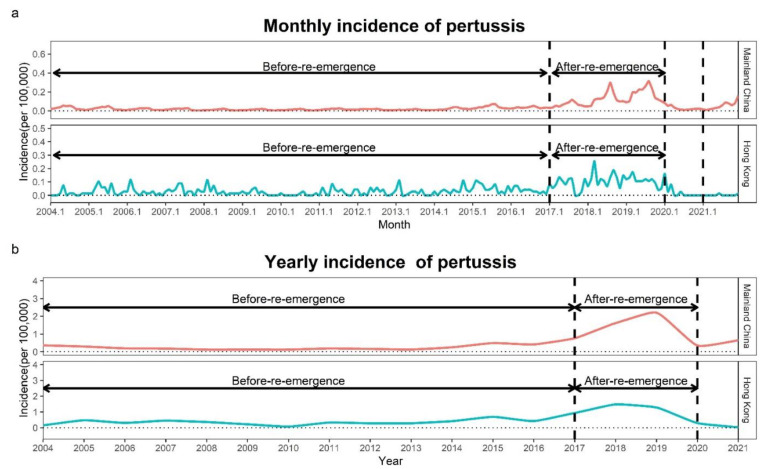
Yearly and monthly incidence rates (1/100,000) of pertussis in the period 2004–2021. (**a**) Monthly incidence of pertussis; (**b**) yearly incidence of pertussis. Before-re-emergence: 2004–2016; after-re-emergence: 2017–2019.

**Figure 3:**
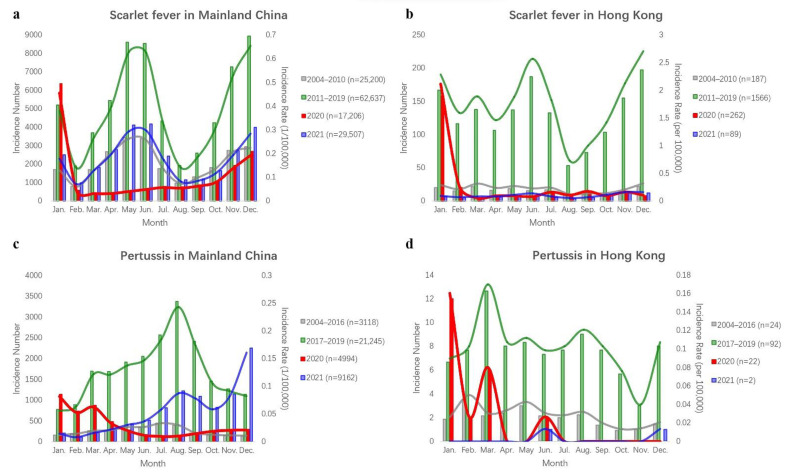
Comparison of monthly incidence rates (1/100,000) of scarlet fever and pertussis. (**a**) Scarlet fever in Mainland China; (**b**) scarlet fever in Hong Kong; (**c**) pertussis in Mainland China; (**d**) pertussis in Hong Kong.

In the period 2004–2019, the total number of cases of scarlet fever was 740,134, with a yearly average of 46,258, and that of pertussis was 104,269, with a yearly average of 6517 in Mainland China. As shown in Figure 1 and Figure 2, increased fluctuations in the incidence of scarlet fever and pertussis were observed in the period 2004–2019, scarlet fever showed a re-emergence trend in the period 2011–2019, and pertussis increased significantly in the period 2017–2019. In 2019, the numbers of cases of the two diseases peaked at 83,028 and 30,727, respectively. Scarlet fever and pertussis have different monthly incidence trends. Scarlet fever has two peaks, mainly occurring from May to June and again in November to January of the following year, while pertussis mainly occurs from July to September. In particular, the monthly incidence of the two diseases decreased, and the seasonality of both diseases disappeared in 2020. In 2021, scarlet fever showed the same trends as before COVID-19, and pertussis showed two peak outbreaks in August and November–December 2021 (Figure 1, Figure 2 and Figure 3). According to the results of the Pearson correlation coefficient test, we found a significant correlation between scarlet fever and pertussis (*r* = 0.33, *p* < 0.001), but neither scarlet fever (*r* = −0.08, *p* = 0.710) nor pertussis (*r* = 0.14, *p* = 0.510) had a significant correlation with COVID-19.

**Figure 4:**
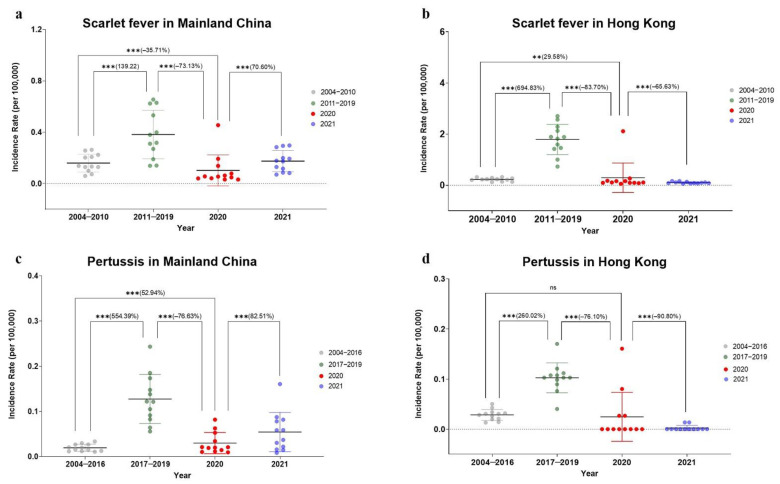
Comparison of yearly incidence rates (1/100,000) of scarlet fever and pertussis. (**a**) Scarlet fever in Mainland China; (**b**) scarlet fever in Hong Kong; (**c**) pertussis in Mainland China; (**d**) pertussis in Hong Kong. The *p* value was computed through two-proportion Z-test; ns: *p* value > 0.05, **: *p* value < 0.01, ***: *p* value < 0.001; the calculated changes are in parentheses, changes = (*x*_1_ − *x*_2_)/*x*_2_ × 100%, *x*_1_: average monthly incidence in 2020, 2021, or after-re-emergence stage (scarlet fever, 2011–2019; pertussis, 2017–2019); *x*_2_: average monthly incidence in before-re-emergence (scarlet fever, 2004–2010; pertussis, 2004–2016), after-re-emergence (scarlet fever, 2011–2019; pertussis, 2017–2019), or 2020.

We compared the changes in the average yearly incidence between the before-COVID-19 stage and the after-COVID-19 stage in Mainland China (Figure 4, Table 1). The number of cases of scarlet fever infection in 2020 and 2021 was 17,206 and 29,507, respectively. The incidence of scarlet fever decreased by 35.71% in 2020 and increased by 9.68 in 2021 compared to that in the before-re-emergence stage (*p* < 0.001), and decreased by 73.13% and 54.15% in 2020 and 2021, respectively, compared to that in the after-re-emergence stage (*p* < 0.001). The number of pertussis infections in 2020 and 2021 was 4994 and 9162, respectively, a 52.94% and 179.12% increase in incidence, respectively, compared to that in the before-re-emergence stage (*p* < 0.001), and a 76.63% and 57.35% decrease in incidence, respectively, compared to that in the after-re-emergence stage (*p* < 0.001). The average yearly incidence of scarlet fever and pertussis in 2021 increased significantly compared to that in 2020, with an increase of 70.60% and 82.51% (*p* < 0.001), respectively. Thus, we observed a rebound in the incidence of scarlet fever and pertussis in 2021, which is already higher than that in the before-re-emergence stage.

### 3.2. Description of the Incidence of Scarlet Fever and Pertussis in Hong Kong

In Hong Kong, the total number of cases of scarlet fever was 15,399 in the period 2004–2019, with an average per year of 962, and that of pertussis was 588, with an average per year of 37. Similar to Mainland China, the number of cases of scarlet fever increased significantly in 2011–2019, and that of pertussis increased significantly in 2017–2019 compared to the previous period in Hong Kong. Scarlet fever peaked with 2353 cases in 2017, whereas pertussis had the highest number of infected cases in 2018, with 110 cases (Figure 1, Figure 2 and Figure 3). There were two peaks of scarlet fever in Hong Kong, mainly from May to June and again from November to March of the following year. The number of cases of pertussis in Hong Kong was relatively small. With 19 cases in March 2018, pertussis reached its peak in the period 2004–2019. In Hong Kong, we observed similar results with Mainland China, with a positive correlation between scarlet fever and pertussis (*r* = 0.42, *p* < 0.001), and neither scarlet fever (*r* = −0.15, *p* = 0.481) nor pertussis (*r* = −0.19, *p* = 0.383) had a significant correlation with COVID-19.

By comparing the changes in incidence of scarlet fever and pertussis before- and after-COVID-19 stage in Hong Kong (Figure 4, Table 2), we observed that, in 2020, the average yearly incidence of scarlet fever increased by 29.58% (*p* = 0.006) compared to that in the before-re-emergence stage and decreased by 83.70% compared to that in the after-re-emergence stage (*p* < 0.001). In 2021, the incidence decreased by 55.47% and 94.40%, respectively, compared to the before- and after-re-emergence stages (*p* < 0.001). The average yearly incidence of pertussis decreased by 92.09% in 2021 compared to the before-re-emergence stage (*p* < 0.001), whereas there was no significant difference in 2020. Compared with the after-re-emergence stage, the incidence decreased by 76.10% in 2020 and 97.80% in 2021 (*p* < 0.001). Compared with 2020, the incidence of scarlet fever and pertussis decreased by 65.63% and 90.80% in 2021 (*p* < 0.001), respectively.

### 3.3. Description of the Difference in the Incidence of Scarlet Fever and Pertussis during the Emergency and Routine Responses to COVID-19 in Mainland China in 2020

In the emergency response stage in Mainland China in 2020, the average monthly incidence of scarlet fever increased by 7.39% (*p* = 0.031) and decreased by 52.85% (*p* < 0.001) compared to that in the before- and after-re-emergence stages, respectively, and in the routine response stage, the average monthly incidence decreased by 51.82% and 80.21% in 2020 compared to that in the before- and after-re-emergence stage, respectively (*p* < 0.001). There was no significant change in the incidence of scarlet fever in the emergency response stage in 2021 compared to 2020, but an increase of 126.84% was observed in the routine response stage. The average monthly incidence of pertussis in the emergency response stage in 2020 increased by 246.13% compared to that in the before-re-emergence stage (*p* < 0.001) and significantly decreased (by 36.31%) compared to that in the after-re-emergence stage (*p* < 0.001), which is due to the low incidence of pertussis in the period 2004–2016 and the significant increase in the period 2017–2019, as shown in Figure 1 and Figure 2. The average monthly incidence of pertussis decreased by 73.90% in 2021 compared to that in 2020 in the emergency response stage (*p* < 0.001). In the routine response stage, the average monthly incidence of pertussis in 2020 decreased by 24.43% (*p* = 0.002) and 89.19% (*p* < 0.001) compared to that in the before- and after-re-emergence stage, respectively. However, the average monthly incidence increased significantly in 2021 compared to that in 2020 in the routine response stage (369.39% increase, *p* < 0.001). Comparing the emergency response stage with the routine response stage in 2020, the average monthly incidence of scarlet fever and pertussis in the routine response stage decreased by 39.96% and 72.74%, respectively (*p* < 0.001, Supplementary Table S1).

### 3.4. Description of the Difference in the Incidence of Scarlet Fever and Pertussis during the Emergency and Routine Responses to COVID-19 in Hong Kong in 2020

In Hong Kong, compared to the same stage in the before- and after-re-emergence stages, the average monthly incidence of scarlet fever increased by 139.36% and decreased by 65.05%, respectively, in the emergency stage in 2020 (*p* < 0.001), while pertussis showed no significant difference. In the 2020 routine stage, scarlet fever and pertussis decreased by 93.15% (*p* < 0.001) and 96.49% (*p* = 0.012), respectively, compared to the same stage in the after-re-emergence stage, with no significant difference compared to the before-re-emergence stage. Compared with the emergency stage in 2020, scarlet fever and pertussis decreased by 87.15% (*p* < 0.001) and 100% (*p* = 0.025), respectively, in the same stage in 2021, while there was no significant difference of two diseases in the routine stage of 2021 compared to 2020. In 2020, scarlet fever and pertussis decreased by 80.69% (*p* < 0.001) and 95.00% (*p* = 0.038, Supplemental Table S1), respectively, in the routine stage compared to the emergency stage 2020.

### 3.5. Comparison of the Observed and Predicted Incidences of Scarlet Fever and Pertussis in Mainland China

**Figure 5:**
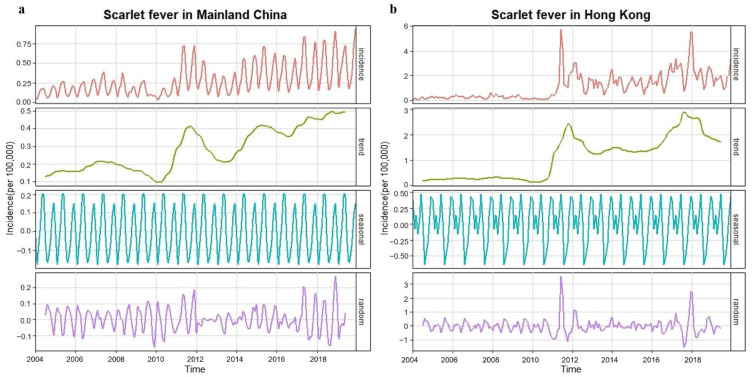
Monthly incidences (1/100,000) in the period 2004–2019 of scarlet fever and long-term trends, seasonal fluctuations, and random fluctuations. (**a**) Scarlet fever in Mainland China; (**b**) scarlet fever in Hong Kong.

**Figure 6:**
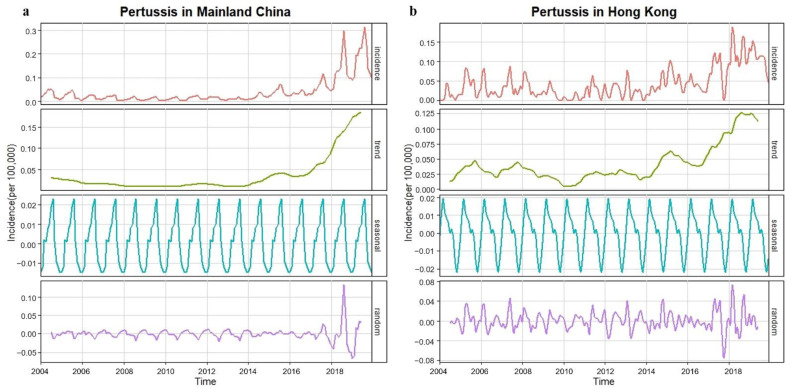
Monthly incidences (1/100,000) in the period 2004–2019 of pertussis and long-term trends, seasonal fluctuations, and random fluctuations. (**a**) Pertussis in Mainland China; (**b**) pertussis in Hong Kong.

We showed the monthly incidences of scarlet fever and pertussis, long-term trend, seasonal fluctuation, and random fluctuation in the period 2004–2019 using the decomposition method (Figure 5 and Figure 6). In mainland China, the incidence of the two diseases showed fluctuating upward trends and significant seasonality. The results of an augmented Dickey–Fuller (ADF) test showed that the data for both diseases tended to be stable after first-order non-seasonal difference (*d* = 1) and first-order seasonal difference (*D* = 1, *S* = 12) (*p* = 0.01). The autocorrelation function (ACF) and partial autocorrelation function (PACF) were used to determine the candidate models (Supplemental Figure S1). We used the Ljung–Box test to confirm that the residual sequence of the models was white noise (Supplementary Figure S2, scarlet fever *p* = 0.90, pertussis *p* = 0.61). Akaike information criterion (AIC) and decision coefficient $R^{2}$ were used to identify the optimal model from all candidate models (Supplemental Table S2). 

**Figure 7:**
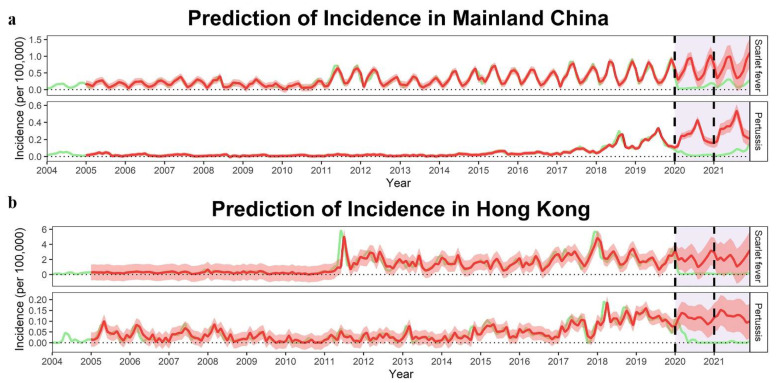
Prediction of the monthly incidence rates (1/100,000) of scarlet fever and pertussis from 2020 to 2021. (**a**) Prediction of incidence in Mainland China; (**b**) prediction of incidence in Hong Kong. Red lines mean fitted and predicted lines; light green lines mean observed ones. The predicted monthly incidences from 2020 to 2021 were generated using ARIMA based on the corresponding values during the period 2004–2019.

Based on the optimal models and morbidity data for the period 2004–2019, the incidence of the two diseases in the absence of COVID-19 from 2020 to 2021 was predicted to be much higher than the actual incidence, as shown in Figure 7. Compared to the predicted incidence, the actual incidence of scarlet fever and pertussis decreased by 72.40% and 67.61% in the emergency stage in 2020, by 87.45% and 94.10% in the routine stage in 2020, and by 74.17% and 82.59% in 2021 (*p* < 0.001, Table 3), respectively.

### 3.6. Comparison of the Observed and Predicted Incidences of Scarlet Fever and Pertussis in Hong Kong

Similar to Mainland China, the incidences of these two diseases in Hong Kong also showed an upward trend of fluctuation in the period 2004–2019, with obvious seasonality (Figure 5 and Figure 6). Using the ARIMA model to predict the monthly incidences of scarlet fever and pertussis in the absence of COVID-19 in 2020–2021, we observed that the predicted incidence of these two diseases in Hong Kong was much higher than the real incidence (Figure 7). The actual average monthly incidence of scarlet fever decreased by 72.17%, 94.05%, and 95.38% compared to the predicted incidence during the emergency stage 2020, the routine stage 2020, and 2021, respectively (*p* < 0.001). There was no significant difference between the predicted and actual incidence of pertussis in the emergency stage 2020, whereas the actual incidence of pertussis in the routine stage 2020 and 2021 decreased by 96.89% (*p* = 0.007) and 98.16% (*p* = 0.003, Table 3), respectively.

## 4. Discussion

This study analyzed the incidence trends of scarlet fever and pertussis in Mainland China and Hong Kong from 2004 to 2021. The incidence of both diseases showed a fluctuating upward trend from 2004 to 2019, with a sudden increase in scarlet fever in 2011–2019 and a sudden increase in pertussis in 2017–2019. However, the incidence of both diseases decreased significantly in 2020 and 2021, leading us to find that non-pharmacological interventions implemented during the COVID-19 pandemic were able to significantly reduce the incidence of scarlet fever and pertussis. In Mainland China, as the epidemic situation gradually improves, COVID-19 prevention and control policies are being relaxed. Although the impact of mild non-pharmacological interventions on the incidence of scarlet fever and pertussis in 2021 was significant, it was weaker than in 2020, thus showing a rebound in the incidence of both diseases compared to 2020. Compared with the before-COVID-19 stage, the average yearly incidence of the two diseases in 2021 was slightly higher than that in the before-re-emergence stage, although it was still lower than that in the after-re-emergence stage. However, in Hong Kong, the incidence of scarlet fever and pertussis decreased by 2021 compared to 2020 due to repeated outbreaks of COVID-19 and tightened prevention and control measures.

Several factors are thought to have contributed to the decreased incidence of scarlet fever and pertussis in 2020. First, the COVID-19 public health control measures implemented in Mainland China and Hong Kong have partially affected the decline in the incidence of both diseases. To control and prevent COVID-19, China adopted containment, suppression, and mitigation strategies, including proactive case detection and management, tracing and isolating close contacts, strict restrictions, or controls on population movement when feasible and appropriate, as well as some personal protection. Since scarlet fever and pertussis are both bacterial diseases that are spread by droplets from coughs and sneezes as COVID-19, intervention measures decreased the exposure to these diseases through decreasing human-to-human transmission by implementing increased social distancing, which reduced the spread of both diseases while controlling the COVID-19 outbreak. Isolating confirmed and suspected cases and social distancing the elderly and vulnerable reduced peak critical care demand and the number of deaths [29]. Further, school closures reduced the spread of respiratory infections among school-age children and adolescents, as schools are a place of high transmission of respiratory infectious diseases [30,31]. Second, the outbreak of COVID-19 in 2020 strained health care services. During the COVID-19 pandemic, fear of contracting COVID-19 and closed-off community management inconvenienced some patients; thus, those with mild symptoms might have sought less or no medical attention, which may understate the incidences of both diseases in the 2020 emergency response stage. However, some symptoms of scarlet fever and pertussis are similar to those of COVID-19, and the diseases may be easier to detect when symptoms are more severe. Third, bed availability, capacity, and the number of health care workers decreased significantly in China during the outbreak of COVID-19 in 2020 and 2021 [32]. These induced decreased admission to hospital for other important infections/diseases. Fourth, people’s living habits changed due to the epidemic: they had to wear face masks when going out and maintain social distancing, which reduced the risk of the spread of these two diseases.

The average monthly incidence of scarlet fever and pertussis in 2021 rebounded compared to 2020 in Mainland China. As the COVID-19 situation improved, the non-pharmacological interventions implemented in Mainland China in 2021 were milder than those implemented in 2020, particularly compared with the emergency stage 2020. If there were no local cases, then, except for wearing masks and using health codes in crowded places, life was a little different from before the epidemic. Thus, the effectiveness of cutting off the transmission route of respiratory diseases was relatively low. After a year of adaptation in 2020 and the successful development and promotion of COVID-19 vaccines, the mobility of the population increased, while personal protective measures decreased, and people gradually relaxed their awareness of protection. In contrast to Mainland China, the incidence of scarlet fever and pertussis in Hong Kong continued to decline in 2021, reaching the lowest value since 2004. This phenomenon can be attributed to the fact that there were several outbreaks of COVID-19 in Hong Kong from 2020 to 2021, and relevant prevention and control policies, such as social distancing and suspending the opening of some public measures, were tightened many times, which reduced person-to-person contact and thus reduced the spread of scarlet fever and pertussis. Further, in 2017–2018, 40% of the cases of pertussis in Hong Kong were infants under 6 months old, and all of them had not completed the primary series of pertussis vaccinations. More than 50% of them were infants less than 2 months old who had not received their first dose of pertussis vaccine [14]. Since 28 June 2020, the pertussis vaccine for pregnant women has been introduced as part of routine antenatal care in Hong Kong, thus providing direct protection for infants in the first few months after birth, before their vaccination has been completed, which is effective at preventing infants from acquiring pertussis [14].

In addition to the impact of the COVID-19 outbreak on the incidence of scarlet fever and pertussis, we analyzed trends in the incidence of these two diseases before the outbreak. In Mainland China and Hong Kong, both diseases showed fluctuating upward trends from 2004 to 2019. The incidence of pertussis increased significantly in the period 2017–2019, while the incidence of scarlet fever increased significantly since 2011, especially in the period 2017–2019, which was the three years with the highest number of infected cases since 2004. According to existing studies, the significant increase in scarlet fever in Mainland China and Hong Kong in 2011 might have been mainly caused by emm12 GAS isolates with multiple antibiotic resistances of tetracycline and macrolide, as well as emm1 GAS isolates [33]. The increased incidence of scarlet fever and pertussis can also be attributed to the natural circulation patterns of both diseases [3,34]. Children are widely vaccinated against pertussis; however, vaccination uptake has not changed the epidemic cycle inherent in the disease, and no vaccine has been developed for scarlet fever. Another possible reason might be that the gradual improvement of the surveillance system for scarlet fever and pertussis in China, as well as the improvement of the surveillance awareness and diagnosis level of medical staff, has increased the detection and reporting rates of both diseases.

This study has several limitations. First, because the data were obtained from the passive monitoring system, the data collection would have been affected by behaviors such as the existing disease diagnosis and medical treatment behavior, which may have resulted in data bias. Second, to reduce the possibility of COVID-19 infection and the requirements of protection policies, some patients with mild symptoms may not seek hospital care, and the actual number of cases of both diseases may be underestimated during the emergency stage of 2020. Third, the incidence of scarlet fever and pertussis is affected by a variety of factors, and the ARIMA model can only be used for time factor analysis; thus, the prediction of future incidence trends may not be very accurate. Nevertheless, this method has been applied in a number of studies and has been proven to be appropriate [24].

## 5. Conclusions

The evidence presented in this study suggests that COVID-19-related non-pharmaceutical measures in Mainland China and Hong Kong significantly helped reduce the spread of scarlet fever and pertussis. These policies can effectively solve the problem of the re-emergence of these two diseases and are worthy of reference for other countries and regions facing this problem. By analyzing the incidence of the two diseases in Mainland China and Hong Kong in 2021, we observed that the incidence of the disease rebounded after the relaxation of COVID-19 epidemic prevention policies. However, compared with the after-re-emergence stage, the incidence of the two diseases still decreased significantly, indicating that relatively mild epidemic prevention policies still played an effective role in preventing the two diseases. As the strict containment and feasible suppression strategies implemented during the COVID-19 pandemic placed a considerable burden on the normal social order of production and life, we surmise that the relatively loose non-drug intervention for the susceptible population during the high incidence period of both diseases effectively reduced the incidence of both diseases. To assist in managing these diseases, balanced infection control and preventive measures are needed in response to outbreaks with prolonged durations or severe outcomes.

## Figures and Tables

**Table 1:** Changes in the average yearly incidences (1/100,000) of scarlet fever and pertussis in 2020 and 2021 compared to previous years in Mainland China.

Diseases	2021 vs. 2020				2021 vs. before-COVID-19 Stage				2020 vs. before-COVID-19 Stage			
	**2021**	**2020**	**Changes (%)**	***p* Value**	**2021**	**Before-Re-Emergence**	**Changes (%)**	***p* Value**	**2020**	**Before-Re-Emergence**	**Changes (%)**	***p* Value**
**Scarlet Fever**	2.10	1.23	70.60	<0.001	2.10	1.92	9.68	<0.001	1.23	1.92	−35.71	<0.001
**Pertussis**	0.65	0.36	82.51	<0.001	0.65	0.23	179.12	<0.001	0.36	0.23	52.94	<0.001
	**2021**	**2020**	**Changes (%)**	***p* Value**	**2021**	**After-Re-Emergence**	**Changes (%)**	***p* Value**	**2020**	**After-Re-Emergence**	**Changes (%)**	***p* Value**
**Scarlet Fever**	2.10	1.23	70.60	<0.001	2.10	4.58	−54.15	<0.001	1.23	4.58	−73.13	<0.001
**Pertussis**	0.65	0.36	82.51	<0.001	0.65	1.53	−57.35	<0.001	0.36	1.53	−76.63	<0.001

Notes: Changes = $\left(x_{1}-x_{2}\right)/x_{2}\times 100\%$, $x_{1}$: average yearly incidence in 2020 or 2021; $x_{2}$: average yearly incidence in before- (scarlet fever, 2004–2010; pertussis, 2004–2016), after-re-emergence (scarlet fever, 2011–2019; pertussis, 2017–2019), or 2020; the *p* value was computed through two-proportion Z-test.

**Table 2:** Changes in the average yearly incidences (1/100,000) of scarlet fever and pertussis in 2020 and 2021 compared to previous years in Hong Kong.

Diseases	2021 vs. 2020				2021 vs. before-COVID-19 stage				2020 vs. before-COVID-19 stage			
	**2021**	**2020**	**Changes (%)**	***p* Value**	**2021**	**Before-Re-Emergence**	**Changes (%)**	***p* Value**	**2020**	**Before-Re-Emergence**	**Changes (%)**	***p* Value**
**Scarlet Fever**	1.20	3.50	−65.63	<0.001	1.20	2.70	−55.47	<0.001	3.50	2.70	29.58	0.006
**Pertussis**	0.03	0.29	−90.80	<0.001	0.03	0.34	−92.09	<0.001	0.29	0.34	−13.96	0.611
	**2021**	**2020**	**Changes (%)**	***p* Value**	**2021**	**After-Re-Emergence**	**Changes (%)**	***p* Value**	**2020**	**After-Re-Emergence**	**Changes (%)**	***p* Value**
**Scarlet Fever**	1.20	3.50	−65.63	<0.001	1.20	21.48	−94.40	<0.001	3.50	21.48	−83.70	<0.001
**Pertussis**	0.03	0.29	−90.80	<0.001	0.03	1.23	−97.80	<0.001	0.29	1.23	−76.10	<0.001

Notes: Changes = $\left(x_{1}-x_{2}\right)/x_{2}\times 100\%$, $x_{1}$: average yearly incidence in 2020 or 2021; $x_{2}$: average yearly incidence in before- (scarlet fever, 2004–2010; pertussis, 2004–2016), after-re-emergence (scarlet fever, 2011–2019; pertussis, 2017–2019), or 2020; the *p* value was computed through two-proportion Z-test.

**Table 3:** Changes in the monthly incidences (1/100,000) of scarlet fever and pertussis between the observed and predicted values from 2020–2021.

Diseases	Emergency Stage (January to April 2020)				Routine Stage (May to December 2020)				2021			
**Mainland China**												
	**Observed**	**Predicted**	**Changes** **(%)**	***p*** **Value**	**Observed**	**Predicted**	**Changes** **(%)**	***p*** **Value**	**Observed**	**Predicted**	**Changes** **(%)**	***p*** **Value**
**Scarlet** **Fever**	0.14	0.51	−72.40	<0.001	0.08	0.67	−87.45	<0.001	0.18	0.68	−74.17	<0.001
**Pertussis**	0.06	0.18	−67.61	<0.001	0.02	0.27	−94.10	<0.001	0.05	0.31	−82.59	<0.001
**Hong Kong**												
	**Observed**	**Predicted**	**Changes** **(%)**	***p*** **Value**	**Observed**	**Predicted**	**Changes** **(%)**	***p*** **Value**	**Observed**	**Predicted**	**Changes** **(%)**	***p*** **Value**
**Scarlet** **Fever**	0.63	2.27	−72.17	<0.001	0.12	2.05	−94.05	<0.001	0.10	2.17	−95.38	<0.001
**Pertussis**	0.07	0.12	−41.95	0.327	0.00	0.11	−96.89	0.007	0.00	0.12	−98.16	0.003

Notes: Changes = $\left(x_{1}-x_{2}\right)/x_{2}\times 100\%$, $x_{1}$: monthly incidence (observed) in 2020–2021; $x_{2}$: monthly incidence (predicted) in 2020–2021; the *p* value was computed using a two-proportion Z-test.

## Data Availability

Publicly available datasets were analyzed in this study. The data can be found here: monthly reported cases of scarlet fever and pertussis in Mainland China http://www.nhc.gov.cn/jkj/s2907/new_list.shtml?tdsourcetag=s_pcqq_aiomsg (accessed on 1 March 2022).; monthly reported cases of scarlet fever and pertussis in Hong Kong: https://www.chp.gov.hk/tc/static/24012.html (accessed on 1 March 2022).

## Supplementary Materials

The following supporting information can be downloaded at: https://www.mdpi.com/article/10.3390/ijerph19169909/s1, Table S1: Changes in the average monthly incidence rates (1/100,000) of scarlet fever and pertussis in the emergency response stage (January to April 2020) and the routine response stage (May to December 2020); Table S2: Parameterization and comparisons of SARIMA models; Figure S1: ACF (**a**,**c**) and PACF (**b**,**d**) of scarlet fever and pertussis after a first-order non-seasonal difference and first-order seasonal difference; Figure S2: Models diagnosis of optimal time series models ARIMA (0,1,3)(1,1,1)12 and ARIMA (4,1,2)(2,1,0)12 of scarlet fever and pertussis.
