# Supplementary material for: Collateral Impact of COVID-19 Prevention Measures on Re-Emergence of Scarlet Fever and Pertussis in Mainland China and Hong Kong China
Source: Int J Environ Res Public Health. 2022 Aug 11;19(16):9909. doi: 10.3390/ijerph19169909 (PMC9407746; doi:10.3390/ijerph19169909)
Supplement: Supplementary file 1 [file ijerph-19-09909-s001.zip › ijerph-1735872-supplementary.pdf]

Collateral Impact of COVID-19 Prevention Measures on Re-Emergence of Scarlet Fever and Pertussis in Mainland China and Hong Kong China

Yiran He, Chenjin Ma, Xiangyu Guo, Jinren Pan, Wangli Xu and Shelan Liu

Supplementary Material

This supplement contains the following Supplementary Tables and Figures to support the manuscript:

Supplemental Table S1: Changes in the average monthly incidence rates (1/100,000) of scarlet fever and pertussis in the emergency response stage (January to April 2020) and the routine response stage (May to December 2020). (pp. 2–3)

Supplemental Table S2: Parameterization and comparisons of SARIMA models. (p. 4)

Supplemental Figure S1. ACF (**a**,**c**) and PACF (**b**,**d**) of scarlet fever and pertussis after a first-order non-seasonal difference and first-order seasonal difference. (p. 5)

Supplemental Figure S2. Models diagnosis of optimal time series models ARIMA (0,1,3)(1,1,1)12 and ARIMA (4,1,2)(2,1,0)12 of scarlet fever and pertussis. (p. 6)

Supplemental Table S1: Changes in the average monthly incidence rates (1/100,000) of scarlet fever and pertussis in the emergency response stage (January to April 2020) and the routine response stage (May to December 2020).

	Emergency stage (January to April 2020) Average monthly incidence (per 100,000)							Routine stage (May to December 2020) Average monthly incidence (per 100,000)							P value (Emergency vs. Routine)	
**Mainland China**																
**Diseases**	**2021**	**2020**	**before-re-emergence**	**2021 vs 2020**		**2020 vs before-re-emergence**		**2021**	**2020**	**before-re-emergence**	**2021 vs 2020**		**2020 vs before-re-emergence**		**Change (%)**	***p* value**
				**Change (%)**	***p* value**	**Change (%)**	***p* value**				**Change (%)**	***p* value**	**Change (%)**	***p* value**		
**Scarlet Fever**	0.14	0.14	0.13	3.07	0.340	7.39	0.031	0.19	0.08	0.17	126.84	<0.001	–51.82	<0.001	–39.96	<0.001
**Pertussis**	0.02	0.06	0.02	–73.90	<0.001	246.13	<0.001	0.07	0.02	0.02	369.39	<0.001	–24.43	0.002	–72.74	<0.001
	**2021**	**2020**	**after-re-emergence**	**2021 vs 2020**		**2020 vs after-re-emergence**		**2021**	**2020**	**after-re-emergence**	**2021 vs 2020**		**2020 vs after-re-emergence**		**Change (%)**	***p* value**
				**Change (%)**	***p* value**	**Change (%)**	***p* value**				**Change (%)**	***p* value**	**Change (%)**	***p* value**		
**Scarlet Fever**	0.14	0.14	0.30	3.07	0.340	–52.85	<0.001	0.19	0.08	0.42	126.84	<0.001	–80.21	<0.001	–39.96	<0.001
**Pertussis**	0.02	0.06	0.09	–73.90	<0.001	–36.31	<0.001	0.07	0.02	0.15	369.39	<0.001	–89.19	<0.001	–72.74	<0.001
**Hong Kong**																
**Diseases**	**2021**	**2020**	**before-re-emergence**	**2021 vs 2020**		**2020 vs before-re-emergence**		**2021**	**2020**	**before-re-emergence**	**2021 vs 2020**		**2020 vs before-re-emergence**		**Change (%)**	***p* value**
				**Change (%)**	***p* value**	**Change (%)**	***p* value**				**Change (%)**	***p* value**	**Change (%)**	***p* value**		
**Scarlet**	0.08	0.63	0.26	–87.15	<0.001	139.36	<0.001	0.11	0.12	0.21	–9.91	0.829	–40.77	0.217	–80.69	<0.001

**Fever**																
**Pertussis**	0.00	0.07	0.04	–100	0.025	89.78	0.397	0.00	0.00	0.03	1.18	0.997	–86.69	0.277	–95.00	0.038
	**2021**	**2020**	**after-re-emergence**	**2021 vs 2020**		**2020 vs after-re-emergence**		**2021**	**2020**	**after-re-emergence**	**2021 vs 2020**		**2020 vs after-re-emergence**		**Change (%)**	***p* value**
				**Change (%)**	***P* value**	**Change (%)**	***p* value**				**Change (%)**	***p* value**	**Change (%)**	***p* value**		
**Scarlet Fever**	0.08	0.63	1.81	–87.15	<0.001	–65.05	<0.001	0.11	0.12	1.78	–9.91	0.829	–93.15	<0.001	–80.69	<0.001
**Pertussis**	0.00	0.07	0.12	–100	0.025	–43.10	0.308	0.00	0.00	0.10	1.18	0.997	–96.49	0.012	–95.00	0.038

Notes: Changes =$(x_1 - x_2)/x_2 \times 100\%$, x_1: average monthly incidence in 2020 or 2021; x_2: average monthly incidence in before- (scarlet fever, 2004–2010; pertussis, 2004–2016), after-re-emergence (scarlet fever, 2011–2019; pertussis, 2017–2019), or 2020, the p value was computed through two-proportion Z-test.

Supplemental Table S2: Parameterization and comparisons of SARIMA models.

Indicators	Parameters #	Parameters1	Parameters2	AIC	AIC1	AIC2	R^2	$R^2$1	$R^2$2
**Mainland China**									
**Forecasting Scarlet Fever Incidence (per 100,000)**	(4,1,2) (2,1,0)	(2,1,2) (2,1,0)	(4,1,3) (2,1,0)	–501·39	–501·28	–501·08	0·93	0·93	0·93
**Forecasting Pertussis Incidence (per 100,000)**	(0,1,3) (1,1,2)	(1,1,3) (0,1,0)	(1,1,3) (0,1,1)	1072·76	–1069·41	–1068·20	0·96	0·96	0·96
**Hong Kong**									
**Forecasting Scarlet Fever Incidence (per 100,000)**	(2,1,1) (0,1,1)	(1,1,2) (0,1,1)	(2,1,3) (2,1,1)	304.87	305.91	306.16	0.77	0.77	0.77
**Forecasting Pertussis Incidence (per 100,000)**	(0,1,2) (1,1,1)	(0,1,2) (0,1,2)	(0,1,3) (1,1,1)	–926.82	–926.46	–926.42	0.83	0.82	0.83

Notes: # The optimized model parameters are listed in the table. These are the top three models with best performance selected from multiple candidate models. In this study, the optimal model was selected according to the principle of minimum AIC and maximum R^2.

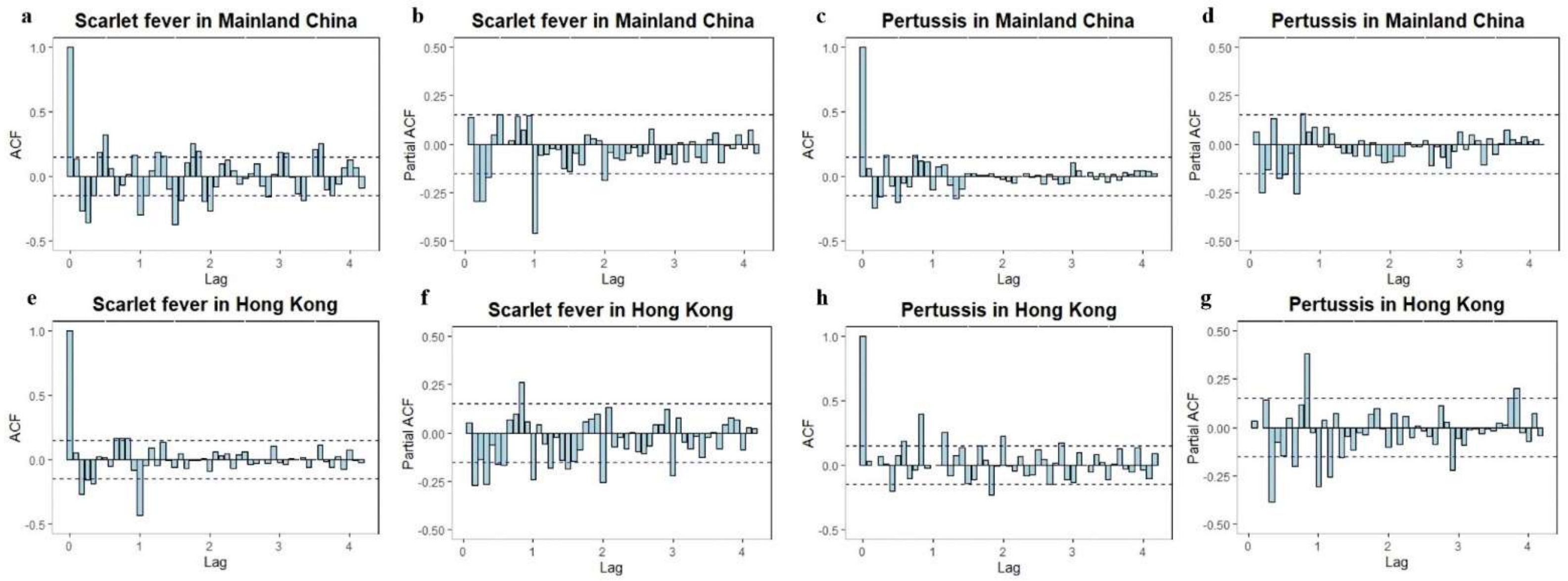

Supplemental Figure S1. ACF (a,c) and PACF (b,d) of scarlet fever and pertussis after a first-order non-seasonal difference and first-order seasonal difference. Notes: ACF, autocorrelation function; PACF, partial autocorrelation functions.

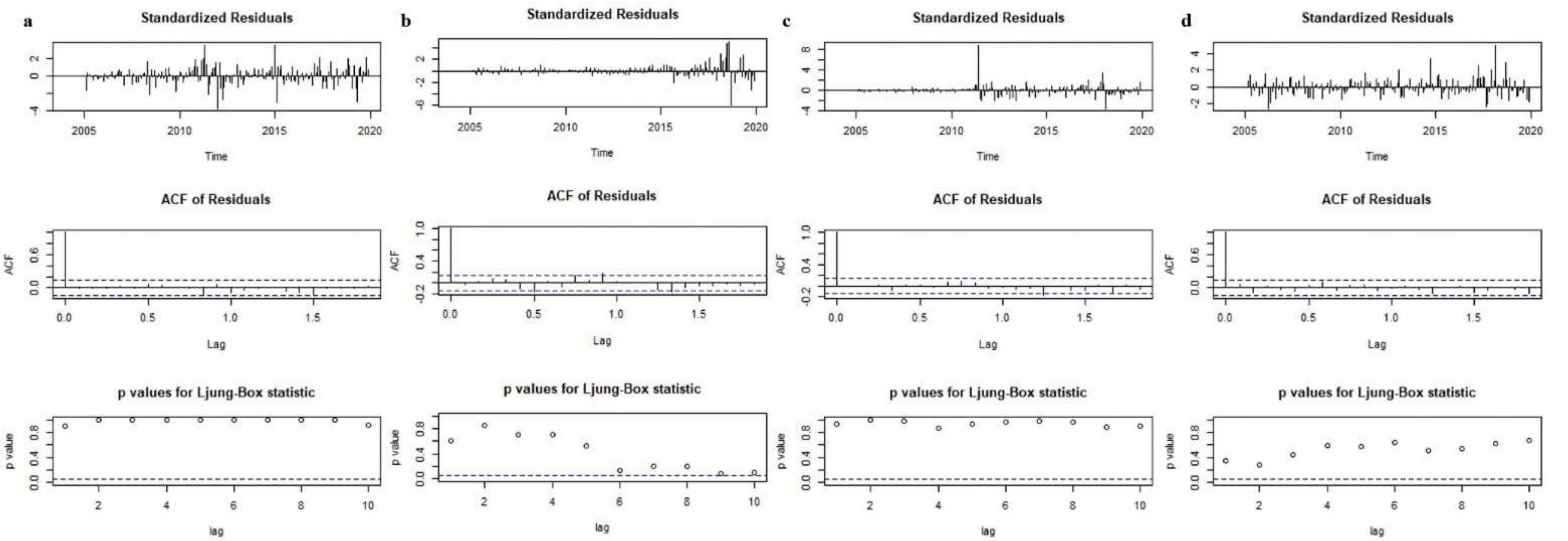

Supplemental Figure S2. Models diagnosis of optimal time series models ARIMA (0,1,3)(1,1,1)12 and ARIMA (4,1,2)(2,1,0)12 of scarlet fever and pertussis. Notes: (a) scarlet fever in Mainland China; (b) pertussis in Mainland China; (c) scarlet fever in Hong Kong; (d) pertussis in Hong Kong; including standardised residual plot, ACF of the errors at various lags and *p* values for Ljung–Box statistic.
